# FDA-Approved AI Solutions in Dental Imaging: A Narrative Review of Applications, Evidence, and Outlook

**DOI:** 10.1016/j.identj.2025.109315

**Published:** 2025-12-19

**Authors:** Sohaib Shujaat, Hend Aljadaan, Hessah Alrashid, Ali Anwar Aboalela, Marryam Riaz

**Affiliations:** a King Abdullah International Medical Research Center, Department of Maxillofacial Surgery and Diagnostic Sciences, College of Dentistry, King Saud Bin Abdulaziz University for Health Sciences, Ministry of National Guard Health Affairs, Riyadh, Saudi Arabia; b King Abdullah International Medical Research Center, College of Dentistry, King Saud Bin Abdulaziz University for Health Sciences, Ministry of National Guard Health Affairs, Riyadh, Saudi Arabia; c Department of Physiology, Azra Naheed Dental College, Superior University, Lahore, Pakistan

**Keywords:** Dental Imaging, Artificial Intelligence, United States Food and Drug Administration, Cloud Computing, Diagnostic Imaging

## Abstract

**Introduction and aims:**

Artificial intelligence (AI) has rapidly transformed dental imaging by enabling automated detection, diagnosis, and analysis of various dental conditions. However, a comprehensive synthesis of United States Food and Drug Administration (FDA)-cleared, clinically validated AI solutions in dental imaging remains limited. This review aims to catalog all standalone, cloud-based dental AI platforms with FDA clearance, highlighting their clinical applications, performance outcomes, and supporting evidence to guide evidence-based integration.

**Methods:**

A two-phase systematic search was conducted. In the first phase, searches of U.S. FDA regulatory databases (510[k], De Novo, and PMA) were performed through July 2025 to identify standalone, cloud-based dental AI imaging devices cleared or authorized for autonomous or semi-autonomous analysis. In the second phase, PubMed, Web of Science, and Google Scholar were systematically searched to retrieve studies assessing the performance or clinical utility of the identified platforms. Two independent reviewers performed data screening and extraction, with discrepancies resolved by a third reviewer.

**Results:**

Thirteen companies were identified as offering twenty-nine FDA-cleared AI products for dental imaging. These solutions addressed diverse clinical tasks, including caries detection, periodontal disease assessment, cephalometric analysis, multi-pathology diagnostics, automated dental charting, and three-dimensional segmentation. Performance outcomes reported by the FDA demonstrated high accuracy, sensitivity, and specificity across most platforms, particularly for caries detection, periodontal disease measurement, and cephalometric analysis. Among these, Relu Creator and WebCeph were supported by the highest number of peer-reviewed publications, whereas several newer platforms lacked independent clinical validation.

**Conclusion:**

Standalone, FDA-cleared AI platforms represent a paradigm shift in dental imaging, providing clinically validated tools for diagnosis, treatment planning, and patient monitoring. By systematically cataloging these solutions, this review delivers an evidence-based reference for clinicians and researchers, supporting informed adoption and identifying areas for future investigation.

## Introduction

Dental imaging is a central pillar of modern dental practice, supporting diagnosis, prevention, and treatment planning across all specialties.^1^ Modalities such as intraoral periapical and bitewing radiography, panoramic imaging, cone-beam computed tomography (CBCT), and clinical photography are indispensable for comprehensive assessment of dentomaxillofacial conditions.^2^^,^^3^ Yet, interpretation of these images remains highly reliant on clinician expertise and subjective judgment, leading to variability in accuracy and consistency.^4^

**Fig. 1:**
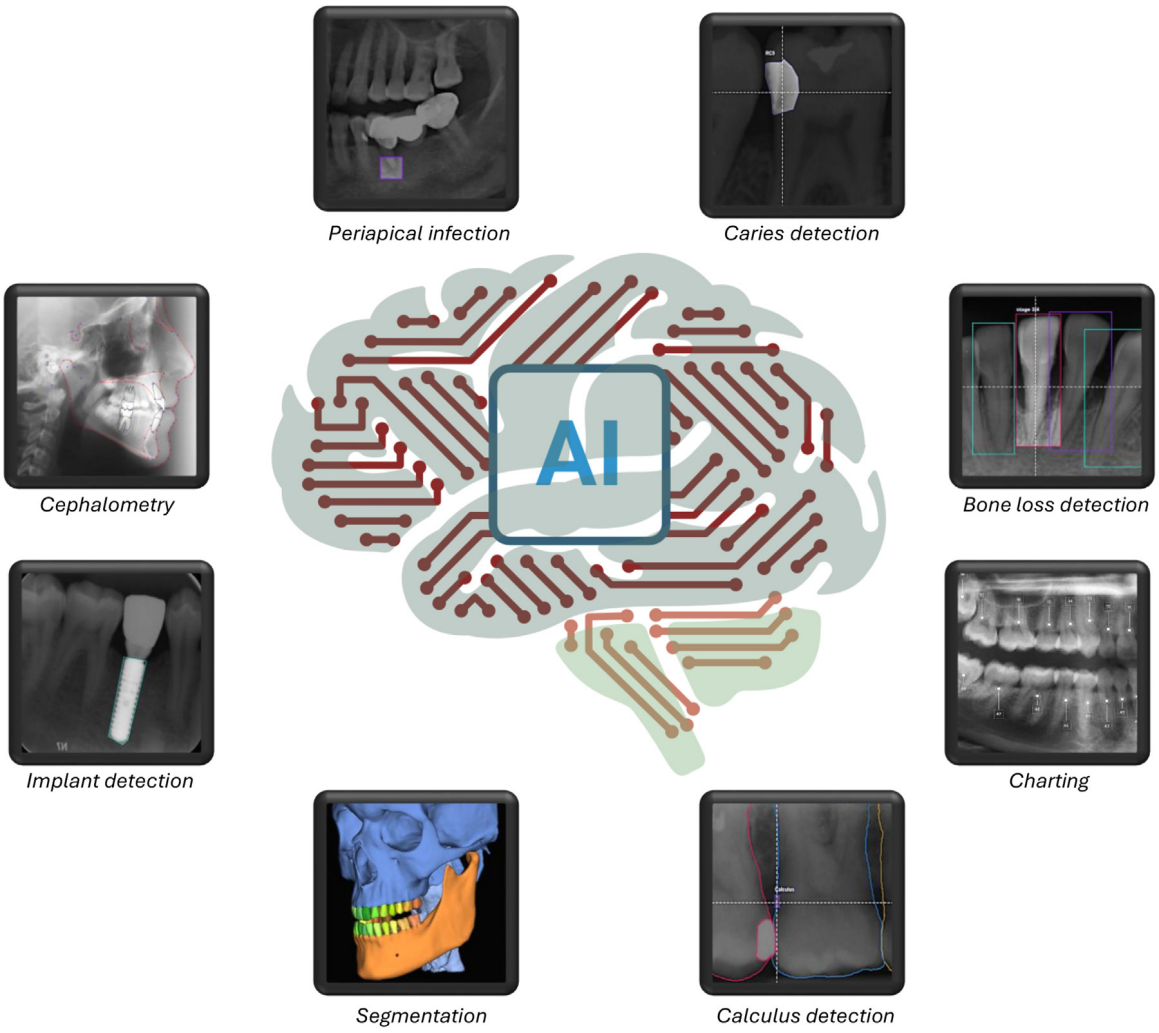
Schematic overview of main clinical domains in dental imaging addressed by AI platforms. Central brain motif represents AI integration in dentistry, with surrounding icons illustrating automated imaging tasks.

**Fig. 2:**
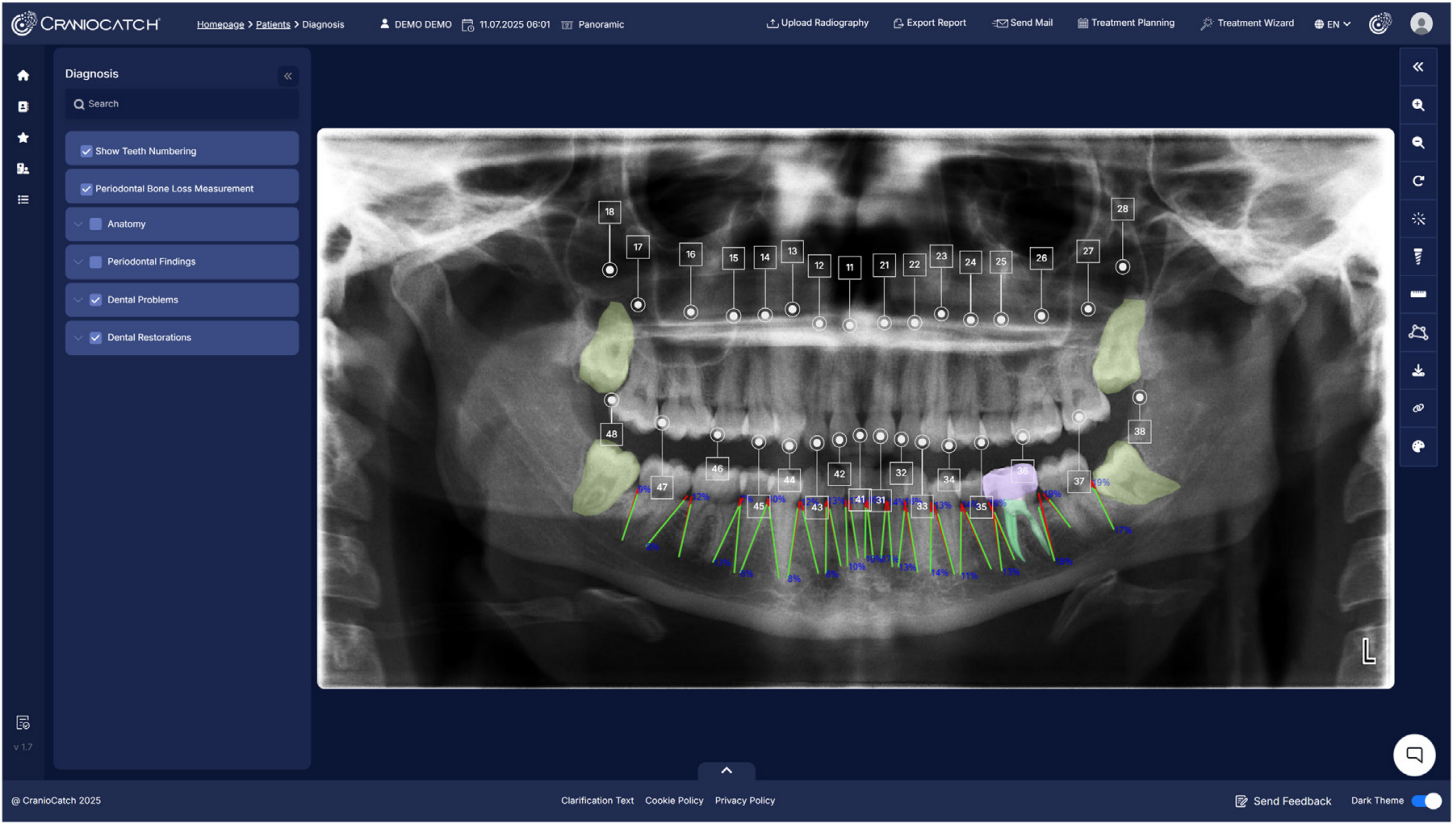
Example of AI-based dental charting using Craniocatch AI platform.

**Fig. 3:**
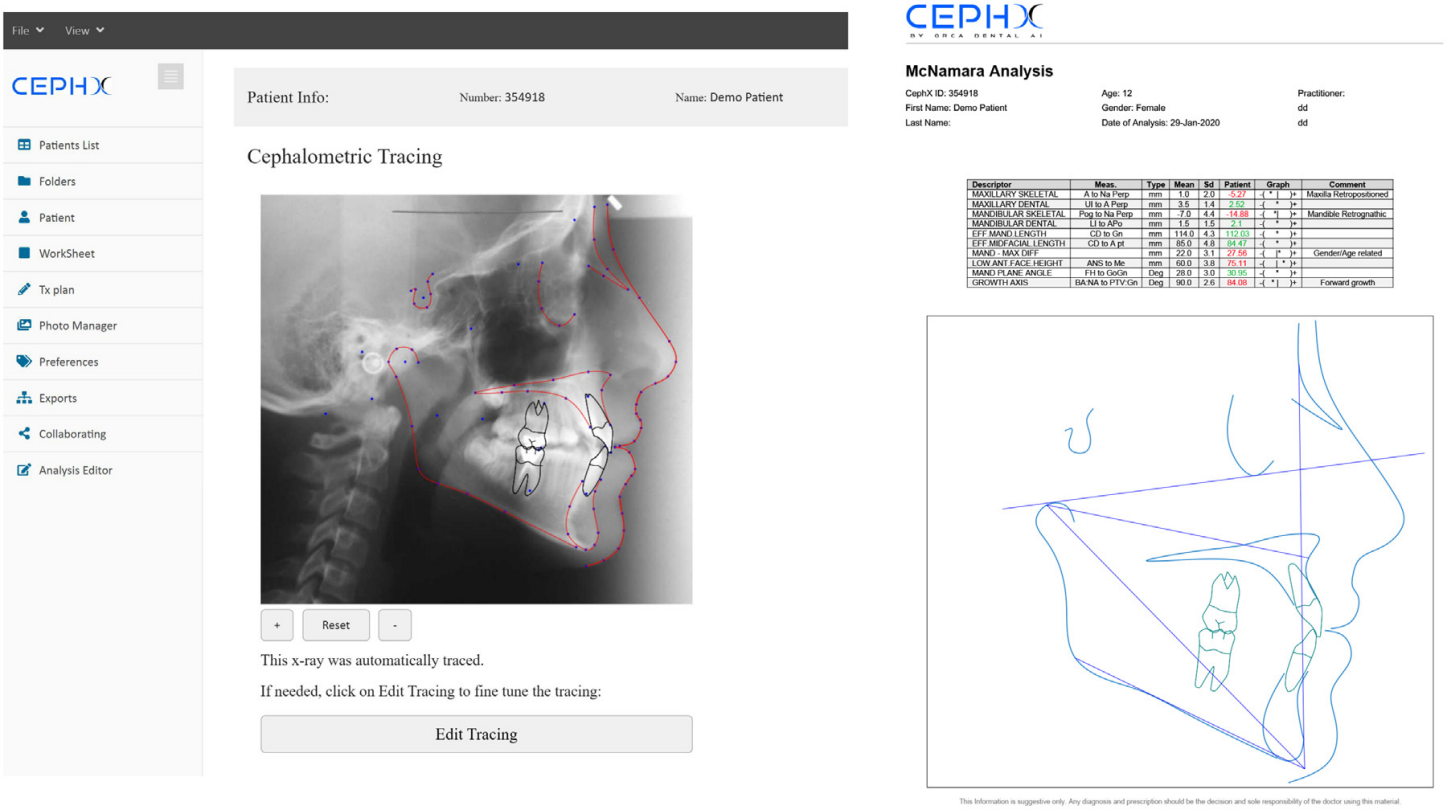
Example of AI-based cephalometric analysis using CephX AI platform.

Artificial intelligence (AI) represents a paradigm shift in dental imaging, addressing longstanding challenges in image interpretation and workflow efficiency.^5^ By leveraging techniques ranging from traditional machine learning to advanced deep learning, AI can automate the extraction, analysis, and interpretation of complex imaging data.^6^ Recent advances have demonstrated that AI-powered tools can detect, segment, classify, and monitor dental conditions based on imaging data with high performance.^7^ The integration of AI in dental imaging spans a broad range of clinical domains (Fig. 1, Fig. 2, Fig. 3). These illustrations provide an overview of AI capabilities, highlighting both the diversity of tasks addressed and examples of automated image analysis in current clinical practice. The adoption of AI is driven by its ability to reduce subjectivity and improve diagnostic sensitivity and specificity.^8^ These technologies support more consistent treatment planning and patient monitoring, extend specialist-level expertise, and enable efficient screening.^9^ Importantly, AI platforms are designed to augment not replace clinical judgment, serving as objective “second readers” to enhance decision-making.^10^

As AI technologies advance, attention is increasingly focused not only on their diagnostic capabilities but also on their integration into clinical dental workflows. A major development in this field is the emergence of standalone, cloud-based dedicated AI platforms specifically designed for dental imaging.^11^ Platforms such as Overjet,^12^ Craniocatch,^13^ VideaHealth,^14^ and Diagnocat^15^ are built around AI as a core component, offering cloud-based, autonomous or semi-autonomous analysis of dental and maxillofacial images. These systems leverage large, expertly annotated datasets and sophisticated machine learning algorithms, including convolutional neural networks (CNNs), to detect radiographic features such as caries, bone loss, periapical pathology, and other clinically relevant findings. Their cloud-based architecture enables remote access through standard web browsers, reduces the need for dedicated local hardware, and allows for centralized updates and algorithm improvements.^¹¹^ In contrast, traditional multipurpose dental software suites, such as CoDiagnostiX,^16^ Planmeca Romexis,^17^ Atomica AI,^18^ DTX Studio,^19^ and Blue Sky Bio,^20^ offer a broad array of digital dentistry tools with integrated AI solutions. While these traditional platforms have incorporated select AI-powered modules (such as virtual modeling or nerve canal identification), they remain primarily clinician-driven, with AI functioning as a supplementary analytic tool.^21^ Typically, these features are processed locally and require more user intervention and ongoing IT support compared to the newer, dedicated AI platforms.^11^ In addition, most integrated AI features in traditional software suites have also not been separately cleared by regulatory authorities as standalone medical devices, in contrast to the explicit regulatory clearance granted to dedicated modules within AI platforms.

This rapid adoption of standalone dedicated AI platforms in healthcare, particularly for diagnostic imaging and treatment planning, has prompted regulatory bodies to adapt their frameworks to ensure the safe and effective use of these technologies.^22^ In the United States, Food and Drug Administration (FDA) has taken a leading role by establishing rigorous standards for the evaluation, clearance, and ongoing surveillance of dedicated AI platforms now transforming dental imaging.^7^ Unlike traditional regulatory oversight, which primarily addresses physical devices and static, locally-installed software, the evaluation of AI-driven platforms introduces unique challenges such as algorithmic transparency, adaptive learning, real-time remote access, and the potential for direct clinical impact.^23^ To address these complexities, the FDA has developed tailored regulatory pathways, including the 510(k) premarket notification for substantially equivalent devices, the De Novo pathway for novel technologies, and Premarket Approval (PMA) for higher-risk products.^24^ Importantly, FDA review of AI platforms now extends beyond technical validation. Manufacturers must demonstrate clinical efficacy, provide transparency in algorithm development, ensure robust performance across diverse patient populations, and implement ongoing post-market surveillance to monitor for safety, performance drift, and potential bias.^25^

FDA clearance serves as a critical benchmark, distinguishing clinically validated AI platforms from experimental technologies and assuring clinicians and patients of a product’s safety, effectiveness, and reliability.^26^ While comprehensive reviews have been conducted in fields such as chest imaging,^27^ neuroradiology,^28^ and abdominal imaging,^29^ to catalogue FDA-cleared AI platforms, similar efforts in dental imaging remain limited. Most reviews in dentistry have often examined experimental algorithms, or focused on general applications of AI in dentistry, without consistently addressing regulatory status or providing an accessible list of AI solutions for clinicians interested in commercially validated solutions.^30^, ^31^, ^32^ To date, there has been no review that catalogues FDA-cleared AI platforms specifically intended for clinical use in dental imaging. The following narrative review aims to fill that gap by presenting, for the first time, a catalogue of dedicated AI products with FDA clearance for dental imaging. The goal is to offer clinicians, researchers, and industry professionals a practical reference to the current portfolio of FDA-approved AI technologies available for use in dental imaging.

## Materials and methods

### Study design and reporting framework

This narrative review was conducted in two sequential phases. Phase 1 systematically identified AI systems cleared by the U.S. FDA for dentomaxillofacial imaging, while Phase 2 retrieved all peer-reviewed publications referencing these FDA-cleared systems. Both phases adhered to the Preferred Reporting Items for Systematic Reviews and Meta-Analyses (PRISMA 2020) guidelines. ^33^

### Phase 1 - Identification of FDA-registered AI systems

#### Data sources and search strategy

Publicly accessible FDA databases, including 510(k), De Novo, and Premarket Approval (PMA),^34^ were comprehensively reviewed to identify AI-based platforms intended for dentomaxillofacial imaging. The 510(k) database was searched using a product-code–based strategy focusing on categories associated with automated image analysis: QIH (automated radiological image processing software), LLZ (system, image processing, radiological), QKB (computer-aided detection/diagnosis software), MYN (diagnostic aid software), and SBC (radiological computer-assisted diagnosis software). Search was limited to radiology and dental panels. The De Novo and PMA databases were screened broadly by panel (radiology and dental) given their limited number of submissions. Duplicate listings across FDA pathways were identified by cross-matching submission numbers, manufacturer names, and device titles, and subsequently removed.

All extracted records were imported into Microsoft Excel (Microsoft Corporation) for data cleaning, deduplication, and eligibility filtering according to the predefined inclusion and exclusion criteria.

#### Eligibility criteria

Platforms were included if they met all of the following conditions: (1) standalone AI solutions with cloud-based deployment, developed for dentomaxillofacial imaging; (2) FDA-cleared or approved under the 510(k), De Novo, or PMA pathways; (3) equipped with autonomous or semi-autonomous AI functionalities supporting diagnostic interpretation, treatment planning, or patient monitoring; and (4) compatible with common dental imaging modalities, including intraoral, panoramic, CBCT, and clinical photography. Exclusion criteria applied to systems functioning solely as embedded AI modules within multipurpose dental software without independent deployment, those lacking regulatory clearance, prototype or investigational systems, and tools designed purely for administrative or nonclinical purposes (eg, data storage or workflow management).

#### Supplementary sources

To ensure completeness, supplementary searches were conducted. The screening involved using commercial and industry resources, including the websites: Crunchbase,^35^ the Institute of Digital Dentistry,^36^ and Dentalcompare.^37^ These platforms were reviewed to identify further companies and products potentially missed in regulatory databases. Searches on Google were also performed to capture additional solutions, using targeted terms such as “dental artificial intelligence,” “cloud-based dental AI,” “AI dental imaging platform,” and “AI dental diagnosis.” All identified entries were cross-verified against the official FDA databases, and only those with confirmed clearance were retained.

#### Data extraction

For each included platform, data were extracted under the following categories:

1. Regulatory Information: Company name, product/module names, FDA clearance number, date, device class, primary product code, submission type, and official website.
2. Clinical and Technical Characteristics: Patient age range, input data type, application area, intended clinical use.
3. FDA Performance Metrics: Sensitivity, specificity, accuracy, area under the curve (AUC), and other indicators reported in FDA documents.

Two reviewers (SS and HA) independently conducted the FDA database search and data extraction. Any discrepancies were resolved through discussion, and unresolved disagreements were adjudicated by a third reviewer (MR) to maintain methodological consistency and minimize bias.

### Phase 2 - Identification of peer-reviewed evidence

#### Data sources and search strategy

Phase 2 targeted all peer-reviewed publications referencing the FDA-cleared AI platforms identified in Phase 1. Searches were conducted in PubMed, Web of Science, and Google Scholar (coverage through July 2025) using keyword combinations related to artificial intelligence, dentistry, and the names of the FDA-cleared platforms. Equivalent keyword logic was applied across databases with minor syntax adjustments where necessary. The general Boolean search string applied was:

(“Adravision” OR “WebCeph” OR “WeDoCeph” OR “Better Diagnostics” OR “Smile Dx” OR “DentalMonitoring” OR “Denti.AI” OR “CephX” OR “Overjet” OR “Pearl” OR “Relu Creator” OR “Velmeni” OR “VideaHealth”) AND (“artificial intelligence” OR “machine learning” OR “deep learning”) AND (“dentistry” OR “dental” OR “oral” OR “radiology” OR “radiograph” OR “imaging” OR “CBCT” OR “panoramic” OR “intraoral” OR “maxillofacial”).

#### Eligibility criteria

Studies were eligible if they evaluated or applied an FDA-cleared commercial AI system. Exclusion criteria comprised of review articles, correspondence, editorials, letters, conference abstracts, and commentaries.

#### Selection process

All retrieved citations were imported into EndNote 21 (Clarivate Analytics), and duplicates were removed. Two reviewers (SS and HA) independently screened titles, abstracts, and full texts, while a third reviewer (MR) adjudicated discrepancies through consensus.

#### Data extraction

Each included article was categorized as either a performance evaluation (quantitative assessment of accuracy, reliability, or performance) or a clinical application report (qualitative description of real-world use without formal validation).

Extracted data included company name, product/module, number of relevant publications, study type (performance evaluation or AI application only), and summary of main findings. The extraction was performed independently by two reviewers (SS and HA). Any discrepancies between the reviewers were resolved through discussion, and unresolved disagreements were referred to a third reviewer (MR) for consensus.

## Results

### Phase 1-Study identification and selection

**Fig. 4:**
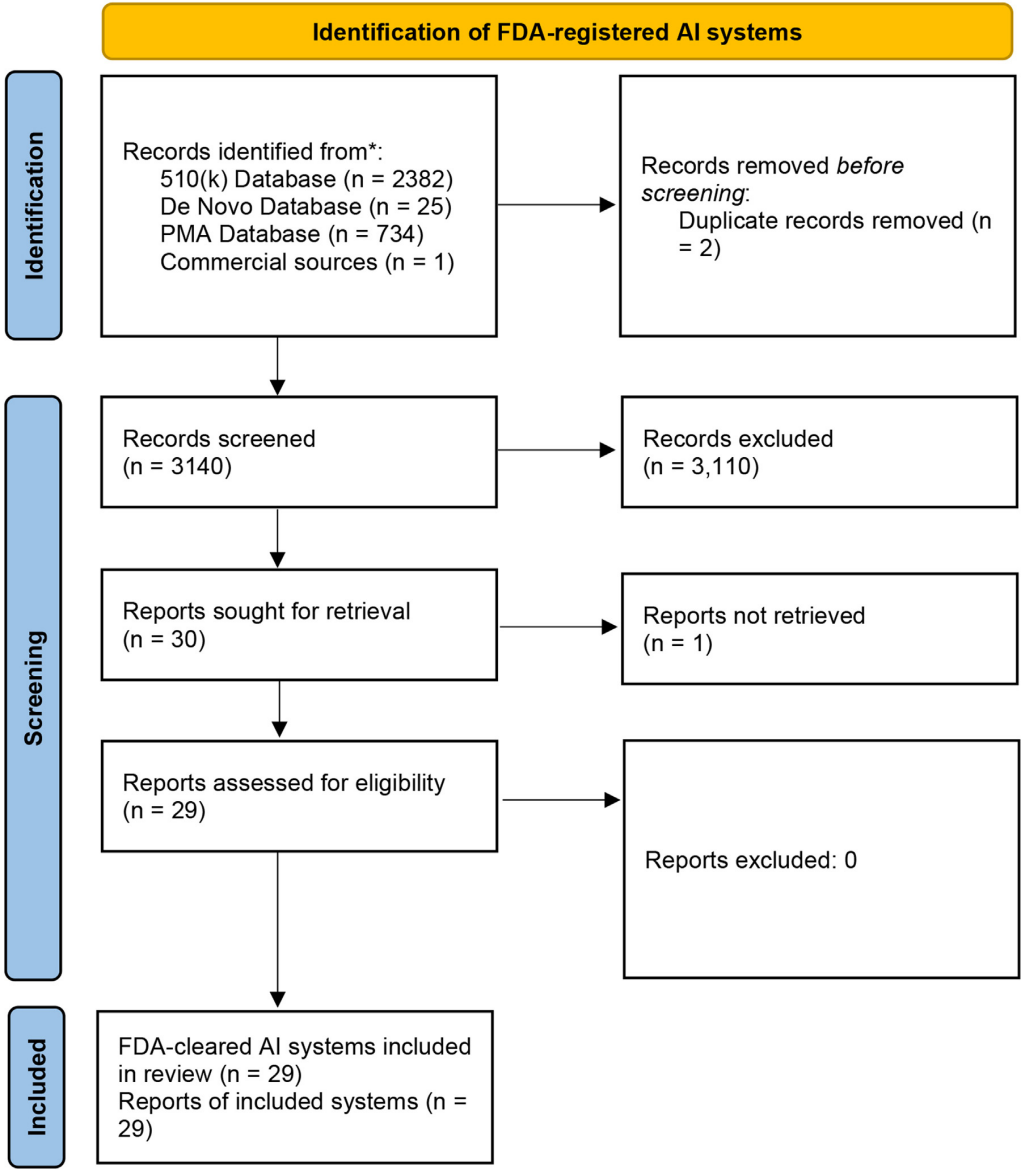
PRISMA flow diagram of the screening and selection process for United States Food and Drug Administration approved dental AI imaging solutions.

Figure 4 illustrates the PRISMA flowchart of FDA regulatory records. A total of 3142 device records were identified from FDA databases [510(k) *n* = 2,382; De Novo *n* = 25; PMA *n* = 734] and one commercial source (*n* = 1). After removal of 2 duplicate listings, 3140 records proceeded to screening. Of these, 3110 were excluded as out of scope (non-dentomaxillofacial imaging, non-AI, embedded modules, administrative-only tools, or not standalone/cloud-deployable). Thirty regulatory submissions were sought for complete decision summaries, of which one was not retrievable. This included, OraQ AI Clinical Decision Support which was listed as FDA approved on the manufacturer’s website. However, no public record of FDA clearance could be verified. Finally, a total of 13 companies were identified as offering FDA-cleared, standalone, cloud-based AI solutions for dental imaging, encompassing 29 distinct products/modules.^38^, ^39^, ^40^, ^41^, ^42^, ^43^, ^44^, ^45^, ^46^, ^47^, ^48^, ^49^, ^50^, ^51^, ^52^, ^53^, ^54^, ^55^, ^56^, ^57^, ^58^, ^59^, ^60^, ^61^, ^62^, ^63^, ^64^, ^65^, ^66^, ^67^

### FDA clearance, regulatory pathways, and product classification

Table 1 provides a list of FDA approved solutions. While many companies maintained broader portfolios of AI-driven solutions, only a subset of their products received FDA clearance for clinical use, rather than their entire commercial lineup. For example, Adravision developed multiple AI tools targeting different dental tasks, but only the Adravision Perio module^38^ had obtained FDA clearance. Similarly, platforms like CephX^46^ and Relu^63^ offered multiple modules within their ecosystem; however, only their core cephalometry and segmentation modules were FDA-cleared, respectively. Conversely, a subset of products developed more extensive FDA-cleared portfolios with multiple modules, reflecting broader clinical capabilities. Overjet led the field with nine FDA-cleared modules covering caries and calculus detection (both pediatric and adult), periapical radiolucency, automated dental charting, and image enhancement.^47^, ^48^, ^49^, ^50^, ^51^, ^52^, ^53^, ^54^, ^55^ Pearl followed with seven FDA-cleared modules, covering a wide range of radiographic applications, including multi-condition detection in bitewing and periapical images, periodontal bone level measurement, segmentation on CBCT images, and specialized modules for pediatric caries and periapical lesion contouring.^56^, ^57^, ^58^, ^59^, ^60^, ^61^, ^62^

**Table 1:** Regulatory and product details of FDA-cleared dental AI platforms.

No.	Company	Product(s) / module(s)	FDA link	FDA 510(k) / De Novo No.	FDA clearance Date	Device class	Primary product Code	Submission type	Website
1	Adravision	Adravision Perio	38	K232440	December 5, 2023	II	QIH	510(k)	adra.ai
2	AssembleCircle	WebCeph	39	K220903	August 17, 2022	II	LLZ	510(k)	webceph.com
3	Audax	WeDoCeph	40	K243005	May 30, 2025	II	QIH	510(k)	wedoceph.com
4	Better Diagnostics	Better diagnostics caries assist (BDCA) Version 1.0	41	K241725	March 11, 2025	II	MYN	510(k)	betterdiagnostics.ai
5	Cube Click	Smile Dx	42	K242437	May 14, 2025	II	MYN	510(k)	smiledx.ai
6	Dental Monitoring SAS	DentalMonitoring	43	DEN230035 (De Novo)	May 17, 2024	II	SBC	De Novo	dentalmonitoring.com
7	Denti.AI	Denti.AI Detect	44	K230144	October 6, 2023	II	MYN	510(k)	denti.ai
		Denti.AI Auto-Chart	45	K222054	November 22, 2022	II	LLZ	510(k)	
8	ORCA Dental AI	CephX Cephalometric Analysis	46	K231396	January 31, 2024	II	QIH	510(k)	cephx.com
9	Overjet	Dental Assist	47	K210187	May 19, 2021	II	LLZ	510(k)	overjet.com
		Caries Assist	48	K212519	May 10, 2022	II	MYN	510(k)	
		Caries Assist	49	K222746	Mar 27, 2023	II	MYN	510(k)	
		Caries Assist - Pediatric	50	K233738	March 4, 2024	II	MYN	510(k)	
		Calculus Assist	51	K220928	December 16, 2022	II	MYN	510(k)	
		Periapical Radiolucency Assist	52	K231678	September 21, 2023	II	MYN	510(k)	
		Charting Assist	53, 54	K233590 (original); K241684 (Predetermined Change Control Plan update)	February 23, 2024 August 27, 2024	II	QIH	510(k)	
		Image Enhancement Assist	55	K241681	September 9, 2024	II	QIH	510(k)	
10	Pearl	Second Opinion	56	K210365	March 4, 2022	II	MYN	510(k)	hellopearl.com
		Second Opinion CS	57	K243234	June 12, 2025	II	MYN	510(k) (Not yet published)	
		Second Opinion CC	58	K242522	January 16, 2025	II	MYN	510(k)	
		Second Opinion Pediatric	59	K243893	May 5, 2025	II	MYN	510(k)	
		Second Opinion BLE	60	K243230	May 9, 2025	II	QIH	510(k)	
		Second Opinion 3D	61	K243989	May 23, 2025	II	QIH	510(k)	
		Second Opinion Periapical Radiolucency Contours	62	K242600	April 11, 2025	II	MYN	510(k)	
11	Relu	Relu Creator	63	K233925	June 13, 2024	II	QIH	510(k)	relu.eu
12	Velmeni	Velmeni for Dentists (V4D)	64	K240003	August 30, 2024	II	MYN	510(k)	velmeni.ai
13	VideaHealth	Videa Dental Assist	65	K232384	December 15, 2023	II	MYN	510(k)	videa.ai
		Videa Perio Assist	66	K223296	February 6, 2023	II	QIH	510(k)	
		Videa Caries Assist	67	K213795	April 21, 2022	II	MYN	510(k)	

FDA clearance dates of products ranged from May 2021 to June 2025. Most approvals clustered between 2022 and 2025, highlighting a recent trend in both technological innovation and regulatory oversight in dental AI. All included products/modules were designated as Class II medical devices, in line with their moderate-risk diagnostic roles. The predominant regulatory pathway was the 510(k) premarket notification, through which all modules were cleared. Only DentalMonitoring platform was approved via the De Novo pathway.^42^ Thereby, recognizing its novelty as a device without a direct predicate.

The most frequently assigned FDA product codes were MYN and QIH, followed by LLZ and SBC:

1. MYN: Designates radiological computer-assisted detection and/or diagnosis software, frequently applied to products intended for caries detection and general dental diagnostics.
2. QIH: Assigned to computer-aided radiology software for maxillofacial and dental imaging, often used for cephalometric analysis, anatomical segmentation, and other advanced imaging functions.
3. LLZ: Used for certain cephalometric analysis and dental charting software modules.
4. SBC: A unique code associated with only DentalMonitoring platform, reflecting its classification through the De Novo regulatory pathway.

### Approved populations and imaging modalities

Table 2 demonstrates the diversity of approved populations and imaging modalities among FDA-cleared dental AI platforms. Some products, such as Adravision Perio (Adravision; ≥22 years) and Cube Click Smile Dx (Cube Click; ≥22 years), were indicated exclusively for adult populations. In contrast, platforms including Overjet Caries Assist-Pediatric (ages 4-11) and Videa Dental Assist (VideaHealth; ≥3 years) were approved for use in pediatric patients. Several modules, such as Pearl Second Opinion BLE (Pearl; ≥12 years) and Overjet Periapical Radiolucency Assist (Overjet; ≥12 years), encompassed both adolescent and adult age groups. In addition, AudaxCeph Cephalogram Analysis (Audax) did not report a specified patient age range in their regulatory documentation.

**Table 2:** Clinical indications and data inputs for FDA-cleared dental AI platforms.

No.	Company	Product(s) / module(s)	Patient age (y)	Input data type	Application area	Intended use
1	Adravision	Adravision Perio	≥22	Bitewing, periapical	Periodontal disease detection	Measurement of mesial and distal alveolar bone levels
2	AssembleCircle	WebCeph	≥8	Lateral cephalometric	Cephalometric analysis	Orthodontic analysis, cephalometric tracing, treatment planning, simulation, and patient consultation
3	Audax	WeDoCeph	Not specified	Lateral cephalometric	Cephalometric analysis	Cephalometric analysis, dental treatment planning, identification of anatomical landmarks, tracing, superimposition, and visual treatment objective generation
4	Better Diagnostics	Better Diagnostics Caries Assist (BDCA) Version 1.0	≥18	Bitewing, periapical	Caries detection	Detection of dental caries (permanent teeth)
5	Cube Click	Smile Dx	≥22	Bitewing, periapical	Multi-condition detection	Detection and segmentation of dental caries, periapical radiolucency, dental restorations, and bone loss (permanent teeth)
6	Dental Monitoring SAS	DentalMonitoring	≥7	Intraoral photographs (smartphone), 3D model scans	Remote orthodontic monitoring	Remote monitoring and assessment of orthodontic treatment and overall oral health (permanent teeth)
7	Denti.AI	Denti.AI Detect	≥22	Bitewing, periapical, panoramic	Multi-condition detection	Detection of dental caries, periapical radiolucency, and bone level measurements (permanent teeth)
		Denti.AI Auto-Chart	≥22	Bitewing, periapical, panoramic	Automated dental charting	Detection and charting of dental structures and restorations (permanent teeth)
8	ORCA Dental AI	CephX Cephalometric Analysis	≥14	Lateral cephalometric	Cephalometric analysis	Detection of cephalometric anatomical landmarks, tracing, simulation, treatment planning, and patient consultation
9	Overjet	Dental Assist	Not specified	Bitewing, periapical	Multi-condition detection	Detection and management of dental caries, bone loss, and periapical radiolucency
		Caries Assist	≥18	Bitewing	Caries detection (adult, 18+)	Detection of dental caries (permanent teeth)
		Caries Assist	≥12	Bitewing, periapical	Caries detection (12+ years)	Detection of dental caries (permanent teeth)
		Caries Assist - Pediatric	4-11	Bitewing, periapical	Pediatric caries detection (4-11 yrs, primary/mixed)	Detection of dental caries (primary, mixed or permanent teeth)
		Calculus Assist	≥18	Bitewing, periapical	Calculus detection	Detection of interproximal dental calculus (permanent teeth)
		Periapical Radiolucency Assist	≥12	periapical	Periapical lesion detection	Detection of periapical radiolucency (permanent teeth)
		Charting Assist	≥5 (bitewing, periapical); not specified (panoramic)	Bitewing, periapical, panoramic	Automated dental charting	Automated charting of tooth anatomy and dental restorations (Bitewing/periapical: primary and/or permanent teeth; panoramic: permanent teeth only)
		Image Enhancement Assist	Not specified	Bitewing, periapical, panoramic	Image enhancement	Enhancement of radiographic image quality, including noise reduction, contrast enhancement, and sharpening
10	Pearl	Second Opinion	≥12	Bitewing, periapical	Multi-condition detection	Detection of dental caries, dental calculus, margin discrepancies, periapical radiolucency, and dental restorations (permanent teeth)
		Second Opinion CS	≥12	Bitewing, periapical	Multi-condition detection	Detection of dental caries, dental calculus, margin discrepancies, periapical radiolucency, and dental restorations (permanent teeth)
		Second Opinion CC	≥19	Bitewing, periapical	Caries detection and segmentation	Detection and segmentation of dental caries (permanent teeth)
		Second Opinion Pediatric	≥4	Bitewing, periapical	Pediatric caries detection	Detection and segmentation of dental caries (primary, mixed or permanent teeth)
		Second Opinion BLE	≥12	Bitewing, periapical	Periodontal disease detection	Measurement of alveolar bone levels (permanent teeth)
		Second Opinion 3D	≥12	Cone-beam computed tomography	3D segmentation a	Segmentation of craniofacial anatomical structures (permanent teeth)
		Second Opinion Periapical Radiolucency Contours	≥12	Periapical Radiographs	Periapical lesion contouring	Detection and segmentation of periapical radiolucencies (permanent teeth)
11	Relu	Relu Creator	Not specified	Cone-beam computed tomography, intraoral Scan, 3D face scan	3D segmentation/registration	Segmentation and registration of craniofacial anatomical structures
12	Velmeni	Velmeni for Dentists (V4D)	≥15	Bitewing, periapical, panoramic	Multi-condition detection	Detection of dental caries, dental restorations, dental prostheses, and dental implants (permanent teeth)
13	VideaHealth	Videa Dental Assist	≥3	Bitewing, periapical, panoramic	Multi-condition detection	Detection and annotation of dental caries, tooth wear, dental calculus, chipped teeth, restorative imperfections, pulp stones, dens invaginatus, periapical radiolucency, widened periodontal ligament space, furcation involvement, crowns, bridges, fillings, and implants (primary, mixed or permanent teeth)
		Videa Perio Assist	≥12	Bitewing, periapical	Periodontal disease detection	Measurement and visualization of mesial/distal bone levels (permanent teeth)
		Videa Caries Assist	≥22	Bitewing	Caries detection	Detection of dental caries (permanent teeth)

Moreover, AI platforms accommodated a range of imaging modalities, reflecting the diversity of clinical needs in dental practice. The majority of solutions were developed to analyze conventional dental radiographs, particularly bitewing and periapical images (eg, Adravision Perio, Better Diagnostics Caries Assist, Pearl Second Opinion). Other platforms, such as Denti.AI Detect and Videa Dental Assist, also supported panoramic radiographs, enabling broader applications in general dental diagnostics. Dedicated cephalometric analysis software including WebCeph, CephX, and AudaxCeph focused on cephalometric radiographs for orthodontic and craniofacial assessment. More advanced systems, like Pearl Second Opinion 3D, were designed to process CBCT data for virtual modeling. Relu Creator went a step further by not only processing CBCT data for segmentation but also automatically aligning CBCT scans with digital impressions and facial scans, providing fully registered three-dimensional (3D) patient models

While most products concentrated on radiographic imaging, a few expanded their capabilities to other modalities. DentalMonitoring, for instance, extended its functionality to include intraoral photographs and 3D model scans.

### Clinical applications

Caries detection was the most common application among FDA-cleared AI platforms, with several modules dedicated to both adult and pediatric populations. Multi-condition detection was frequently supported, enabling identification of caries, restorations, periapical radiolucency, bone loss, calculus, prostheses, and implants. Periodontal disease detection was addressed by modules providing automated measurement of alveolar bone levels, while cephalometric analysis for orthodontic planning was offered by dedicated cephalometric platforms. Additional functionalities included cephalometric analysis, automated dental charting, periapical lesion segmentation, and advanced 3D imaging, with segmentation and registration of craniofacial structures available in Relu Creator and 3D segmentation in Pearl Second Opinion 3D. Remote orthodontic monitoring using intraoral photographs and 3D models was enabled by DentalMonitoring (Table 2).

### FDA based performance outcomes

Table 3 summarizes the performance validation of AI platforms, including results from bench testing and multi-reader evaluations, demonstrating that products met or exceeded regulatory standards for accuracy and reliability.

**Table 3:** Performance metrics and validation outcomes of FDA-cleared dental AI platforms.

No.	Company	Product(s) / module(s)	Performance metrics (FDA)	Performance results (FDA)
1	Adravision	Adravision Perio	OKS Assessment: precision, recall length measurement: sensitivity, specificity, mae tooth number classification accuracy	OKS Assessment: • BW: Precision 91.0%, Recall 94.0% • PA: Precision 84.8%, Recall 89.3% Length Measurement: • BW: Sensitivity 90.7%, Specificity 94.3%, MAE 0.434 mm • PA: Sensitivity 92.5%, Specificity 86.8%, MAE 0.504 mm Tooth Number Accuracy: 91.8% (OKS), 93.2% (Length)
2	AssembleCircle	WebCeph	Software verification/validation measurement accuracy test System-level test	Device passed all tests based on predetermined pass/fail criteria. No quantitative metrics provided in the FDA summary.
3	Audax	WeDoCeph	Automatic Landmark Detection: mean radial error (mre, mm) software verification & validation	• MRE: Lateral ≤1.5 mm (passed), Frontal ≤2.5 mm (passed) • All validation and verification passed per FDA and international standards.
4	Better Diagnostics	Better Diagnostics Caries Assist (BDCA) Version 1.0	Surface/image: sensitivity, specificity	Surface-level: • Sensitivity BW 89.2%, PA 88.2% • Specificity BW 99.5%, PA 99.1% Image-level: • Sensitivity BW 81.0% (conservative), 91.9% (optimistic) • Specificity 98.4% Reader Study: Statistically significant improvement in diagnostic performance (aided vs. unaided).
5	Cube Click	Smile Dx	Dice, sensitivity, standalone & MRMC clinical evaluation	• Caries: Dice 0.74, Sensitivity 88.3% • PA radiolucency: Dice 0.77, Sensitivity 86.1% • Bone loss: Accurate segmentation and measurement in clinical study.
6	Dental Monitoring SAS	DentalMonitoring	Clinical performance (accuracy & precision of algorithms) Nonclinical software verification, validation, usability, biocompatibility	Required to meet FDA special controls: passed clinical and nonclinical validation; specific metrics not disclosed in summary.
7	Denti.AI	Denti.AI Detect	wAFROC AUC, sensitivity, specificity, MAE	• wAFROC AUC: 0.737 • BW: Sensitivity 98.1%, Specificity 93%, MAE 0.513mm • PA: Sensitivity 98.2%, Specificity 88.5%, MAE 0.572 mm Reader Study: significant improvement with aid (*P* = .029).
		Denti.AI Auto-Chart	Sensitivity, PPV, classification accuracy, manual charting reduction rate	• Teeth Detection Sensitivity: 97.4% • PPV: 99.6% • Numbering Accuracy: 85.9% • Restorative Sensitivity: 88.5%, Specificity: 98.3% • Filling-by-Type Accuracy: 98.0% • Manual Charting Reduction Rate: 71.2%
8	ORCA Dental AI	CephX cephalometric analysis	AI landmark detection vs. manual (21 landmarks) % within 2.0 mm of manual expert points	• 99% of landmarks within 2.0 mm margin • Exceeded predefined acceptance (≥85% passed) • Strong reliability & precision • All software validation passed
9	Overjet	Dental assist	Average precision, average recall (software and clinical testing) Interreader agreement	• BW: Precision 91.5%, Recall 93.1% • PA: Precision 93.7%, Recall 95.7% • Interreader agreement: 95.7% • Consistent performance across tooth gender, arch, and jaw
		Caries assist	Standalone: Sensitivity, Specificity, Dice Reader: Sensitivity, Specificity, AFROC	Standalone: • Sensitivity 72.0% (62.9%-81.1%), Specificity 98.1% (97.7-98.5%) • Primary caries: 74.4%, Secondary: 62.5% • Dice (primary): 0.69, (secondary): 0.75 Reader: • Sensitivity improved from 57.9% to 76.2%, Specificity decreased from 99.3% to 98.4% (assisted vs. unassisted) • AFROC AUC improved by 0.057 (statistically significant); Specificity decreased <1%
		Caries assist	Standalone: sensitivity, specificity, Dice Reader: sensitivity, specificity, wAFROC	• BW: Sensitivity 76.6%, Specificity 99.1% • PA: Sensitivity 79.4%, Specificity 99.4% • Dice (BW): Primary 0.77, Secondary 0.73 • Dice (PA): Primary 0.79, Secondary 0.79 Reader: • Sensitivity improved (BW: 64.6%→78.5%, PA: 65.6%→79.0%), Specificity decreased slightly (∼0.4%) • wAFROC AUC improved (BW: +0.055, PA: +0.050), both significant
		Caries assist - pediatric	Standalone: Sensitivity, Specificity, Dice Reader: Sensitivity, Specificity, wAFROC	Standalone: • Tooth-level Sensitivity 83.9% (95% CI: 0.816-0.860), Specificity 97.5% (0.971-0.979), Dice 79.0% Reader: • Tooth-level Sensitivity improved by 11.8%, Specificity decreased by 1.1% • wAFROC AUC improved by 7.5%, all statistically significant
		Calculus assist	Standalone: sensitivity, specificity, AFROC AUC reader: sensitivity	Standalone: • BW Sensitivity 74.1%, Specificity 99.4% • PA Sensitivity 72.9%, Specificity 99.6% • BW AFROC AUC: 0.859 • PA AFROC AUC: 0.867 • Subgroup: Results similar by age/gender/site Reader: • Superiority (increased sensitivity with aid)
		Periapical radiolucency assist	Standalone: sensitivity, specificity MRMC Reader Study: ROC-AUC improvement, sensitivity	Standalone: • Image-level Sensitivity: 89.8% (95% CI: 0.847, 0.914), Specificity: 84.2% (95% CI: 0.810, 0.847) • Polygon-level Sensitivity: 66.4% (95% CI: 0.615, 0.711) Sensor breakdown: • Dexis Sensitivity: 86.7%, Specificity: 88.5% • e2v Sensitivity: 86.1%, Specificity: 80.4% • Gendex Sensitivity: 88.9%, Specificity: 79.3% • Schick Sensitivity: 90.8%, Specificity: 89.1% MRMC Reader: • ROC-AUC improved by 4.8% (stat. sig., *P* < .001) • Reader sensitivity improved by 13.6%; Specificity decreased by 7.1%
		Charting assist	Standalone: sensitivity, specificity, dice, manual charting reduction tooth numbering accuracy	Standalone Performance: • Tooth-level sensitivity (Past restorative): 88.3% (86.6%-90.1%) • Tooth-level sensitivity (Anatomy): 95.9% (95.1%-96.5%) • Specificity (Fillings): 98.6% (98.3%-99.0%) • Specificity (root canal treatment): 99.9% (99.8%-100%) • Specificity (Crown): 99.4% (99.2%-99.6%) • Specificity (Implant): 99.8% (99.7%-99.9%) • Dice: Anatomy 0.836, restorations 0.918 • Manual charting reduction: 80.5% • Tooth numbering accuracy: BW 98.9%, PA 96.9%, Pano 99.2% • Subgroup analysis: consistent performance across gender, age, geography, sensor type
		Image enhancement assist	Not required for enhancement-only tools (no direct detection)	Software enhances BW/PA (noise reduction), BW/PA/Pano (contrast/sharpness); Does not alter AI findings/results; can be toggled on/off by user.
10	Pearl	Second opinion	wAFROC-FOM, Sensitivity, FPPI, Jaccard Index	Standalone Sensitivity: 76.4%-89.8% (across pathologies) FPPI: 0.46-4.85 Aided Reader wAFROC-FOM: 0.758 (vs. unaided 0.740, *P* = .0062) Statistically significant aided-reader improvement in detection accuracy for all tested pathologies.
		Second opinion CS	Not yet published by FDA	Not yet published by FDA.
		Second opinion CC	Standalone sensitivity, Dice, wAFROC-FOM, HR-ROC-AUC	• Standalone sensitivity: 90% (87%-94%) • FPPI: 1.34 (1.20-1.48) • Dice (segmentation accuracy): 0.73 (0.71-0.75) • wAFROC-FOM: 0.81 (0.77-0.85) • HR-ROC-AUC: 0.88 (0.85-0.91) Noninferior to original Second Opinion; exceeds prespecified acceptance criteria.
		Second Opinion Pediatric	Standalone sensitivity, FPPI, Dice, wAFROC-FOM, HR-ROC-AUC	• Standalone sensitivity: 87% (0.84-0.90) • FPPI: 1.22 (1.14-1.30) • Dice (segmentation accuracy): 0.76 (0.75-0.77) • wAFROC-FOM: 0.86 (0.84-0.88) • HR-ROC-AUC: 0.94 (0.93-0.96) Sensitivity significantly >75% in BW and PA images.
		Second opinion BLE	Precision, recall, mean absolute difference (mm)	• BW: Precision 87%, Recall 91%, Mean absolute diff 0.86 mm • PA: Precision 87%, Recall 87%, Mean absolute diff 0.45 mm All results exceeded FDA-accepted thresholds (precision/recall >82%, mean absolute diff <1.5 mm).
		Second opinion 3D	Dice similarity coefficient (per anatomy)	• Dentition: 0.86 (0.83-0.89) • Maxilla: 0.91 (0.91-0.92) • Mandible: 0.97 (0.97-0.97) • Mandibular Canal: 0.76 (0.74-0.78) • Sinus: 0.97 (0.97-0.98) • Nasal: 0.90 (0.89-0.91) • Airway: 0.95 (0.94-0.96) All results statistically significant (p<0.000001).
		second opinion periapical radiolucency contours	wAFROC-FOM, HR-ROC-AUC, Lesion-level Sensitivity, FPPI	• wAFROC-FOM: 0.85 (95% CI: 0.81-0.89) • HR-ROC-AUC: 0.93 (0.90-0.96) • Lesion-level sensitivity: 77% (69-84%) • FPPI: 0.28 (0.23-0.33) Noninferior to original Second Opinion and Overjet; similar efficacy across imaging devices, regions, and ages.
11	Relu	Relu Creator	Software verification & validation	Device passed all verification & validation tests for intended use; supports 3D modeling, segmentation & simulation for planning; no clinical performance data.
12	Velmeni	Velmeni for dentists (V4D)	Standalone: Sensitivity, Specificity, Dice Reader: Sensitivity, Specificity, wAFROC	Standalone (lesion-level sensitivity): • BW: Caries 72.8%, Prosthesis 92.1%, Implant 81.1%, Restoration 88.1% • PA: Caries 70.6%, Prosthesis 81.0%, Implant 94.5%, Restoration 76.8% • Pano: Caries 68.3%, Prosthesis 74.5%, Implant 79.6%, Restoration 72.6% • Dice scores: Caries 82%, Prosthesis 97%, Implant 94%, Restoration 90% (BW); similar for PA/Pano Reader study: Significant improvement in sensitivity/accuracy for all features (eg, caries BW aided: 80.3% vs unaided 67.5%; PA aided: 73.4% vs unaided 48.7%).
13	VideaHealth	Videa dental assist	Sensitivity, specificity, AFROC FOM	• Bench: All indications met acceptance criteria (except caries specificity) • Clinical Reader Study: All aided indications improved AFROC FOM (eg, attrition: +0.171/28.5%, calculus: +0.163/23%, caries: +0.024/4.3%), all *P* < .01 • Pediatric + PA validated.
		Videa perio assist	Sensitivity, specificity, mean absolute error	• Bench: Recall 94.4%/91.9%, Precision 84.3%/79.1% (BW/PA) • Clinical: Sensitivity 92.8%/88.3%, Specificity 89.4%/87.0%, Mean absolute error <1.5mm • Subgroups (sensor/patient age) met criteria except BW specificity for Sirona; no safety/effectiveness concerns.
		Videa caries assist	AFROC FOM, Sensitivity, PPV, FPPI	• Standalone: FOM 0.740 (0.721-0.760), Sensitivity 70.8%, PPV 59.5% • Lesion-based: Sensitivity 73.6%, PPV 64.9% • Clinical Reader Study: FOM (aided) 0.739, FOM (unaided) 0.667, Δ0.072 (*P* < .0001), improved sensitivity for all readers aided vs unaided.

AFROC, Alternative Free-response Receiver Operating Characteristic; AUC, Area Under the Curve; BW, Bitewing; CI, Confidence Interval; FPPI, False Positives Per Image; HR-ROC-AUC, Hierarchical Receiver Operating Characteristic Area Under Curve; MAE, Mean Absolute Error; MRE, Mean Radial Error; MRMC, Multi-Reader Multi-Case; OKS, Overall Keypoint Similarity; PA, Periapical; Pano, Panoramic; PPV, Positive Predictive Value; ROC-AUC, Receiver Operating Characteristic Area Under Curve; wAFROC, Weighted Alternative Free-response Receiver Operating Characteristic; wAFROC-FOM, Weighted AFROC Figure of Merit.

#### Periodontal disease detection

Platforms developed for periodontal disease detection, such as Adravision Perio, Pearl Second Opinion BLE, and Videa Perio Assist, demonstrated robust quantitative accuracy. Adravision Perio achieved bitewing precision and recall rates of 91.0% and 94.0%, respectively, with sensitivity and specificity exceeding 90% for bone level measurement and mean absolute errors (MAE) well below 1 mm. Pearl Second Opinion BLE demonstrated precision and recall rates of 87% and 91%, with measurement errors consistently under 1.5 mm, exceeding FDA-accepted thresholds. Videa Perio Assist showed similar performance, with recall and precision of 94.4% and 84.3% for bitewing images and clinical sensitivity of 92.8% for bone level assessment.

#### Caries detection

Better Diagnostics Caries Assist reported surface-level sensitivity and specificity of 89.2% and 99.5% (bitewing) and significant improvements in diagnostic accuracy with AI assistance. Overjet Caries Assist achieved sensitivities of 72% to 79.4% and specificities above 98% across standalone and reader studies, with segmentation accuracy (Dice coefficient) reaching up to 0.79. Pediatric-specific modules such as Overjet Caries Assist-Pediatric and Pearl Second Opinion Pediatric demonstrated sensitivities above 80% and specificities exceeding 97%, supporting clinical utility in younger age groups. Videa Caries Assist similarly reported sensitivity of 70.8% and significant gains in reader diagnostic performance.

#### Cephalometric analysis and landmark identification

Cephalometric analysis solutions, including WebCeph, AudaxCeph, and CephX, met or surpassed international standards for anatomical landmark detection and tracing. AudaxCeph achieved mean radial errors of ≤1.5 mm for lateral and ≤2.5 mm for frontal cephalograms, with all validation thresholds met. CephX achieved 99% of landmarks within a 2 mm margin of expert annotation, indicating strong reliability and precision. While WebCeph did not report quantitative metrics, all required validation and software accuracy assessments were completed as per FDA submissions.

#### Multi-condition detection and automated charting

Platforms such as Overjet Dental Assist, Denti.AI Detect, Cube Click Smile Dx, Velmeni for Dentists (V4D), and Videa Dental Assist provided multi-condition detection for findings including caries, restorations, bone loss, and calculus, often coupled with automated charting functionalities. Overjet Dental Assist reported precision and recall exceeding 91% and 93%, with interreader agreement of 95.7%. Denti.AI Auto-Chart achieved teeth detection sensitivity of 97.4%, positive predictive value (PPV) of 99.6%, and a manual charting reduction rate of 71.2%. Velmeni reported lesion-level sensitivity for various conditions ranging from 68% to 94.5%, with segmentation Dice coefficients up to 0.97. All platforms demonstrated consistent performance improvements in multi-reader studies, highlighting enhanced clinician accuracy and workflow efficiency with AI integration.

#### Remote orthodontic monitoring and clinical decision support

DentalMonitoring passed all required clinical and nonclinical validation per FDA standards for remote orthodontic monitoring using intraoral photographs and 3D model scans, though specific quantitative performance metrics were not disclosed.

#### 3D segmentation and advanced image processing

Advanced modules such as Relu Creator and Pearl Second Opinion 3D enabled automated 3D segmentation in dental imaging workflows. Relu Creator also supported the registration of CBCT, intraoral, and facial scans for comprehensive image management and treatment planning and passed all required software verification and validation for segmentation and registration. Pearl Second Opinion 3D, in contrast, demonstrated high segmentation accuracy, with Dice coefficients ranging from 0.76 to 0.97 across various anatomical regions.

#### Periapical lesion detection and contouring

Modules targeting periapical lesion detection, such as Overjet Periapical Radiolucency Assist and Pearl Second Opinion Periapical Radiolucency Contours, delivered strong standalone performance. Overjet’s tool reported image-level sensitivity of 89.8% and specificity of 84.2%, with multi-reader studies confirming significant improvements in ROC-AUC and reader sensitivity. Pearl’s module achieved lesion-level sensitivity of 77% and ROC-AUC of 0.93, with noninferiority to other platforms and consistent results across imaging devices and populations.

#### Image enhancement

Overjet Image Enhancement Assist offered automated image quality improvements such as noise reduction and contrast enhancement on bitewing, periapical, and panoramic radiographs. As enhancement-only tools, this module was not evaluated for diagnostic accuracy but enhances interpretability of clinical images without altering underlying diagnostic results.

### Phase 2- Study identification and selection

**Fig. 5:**
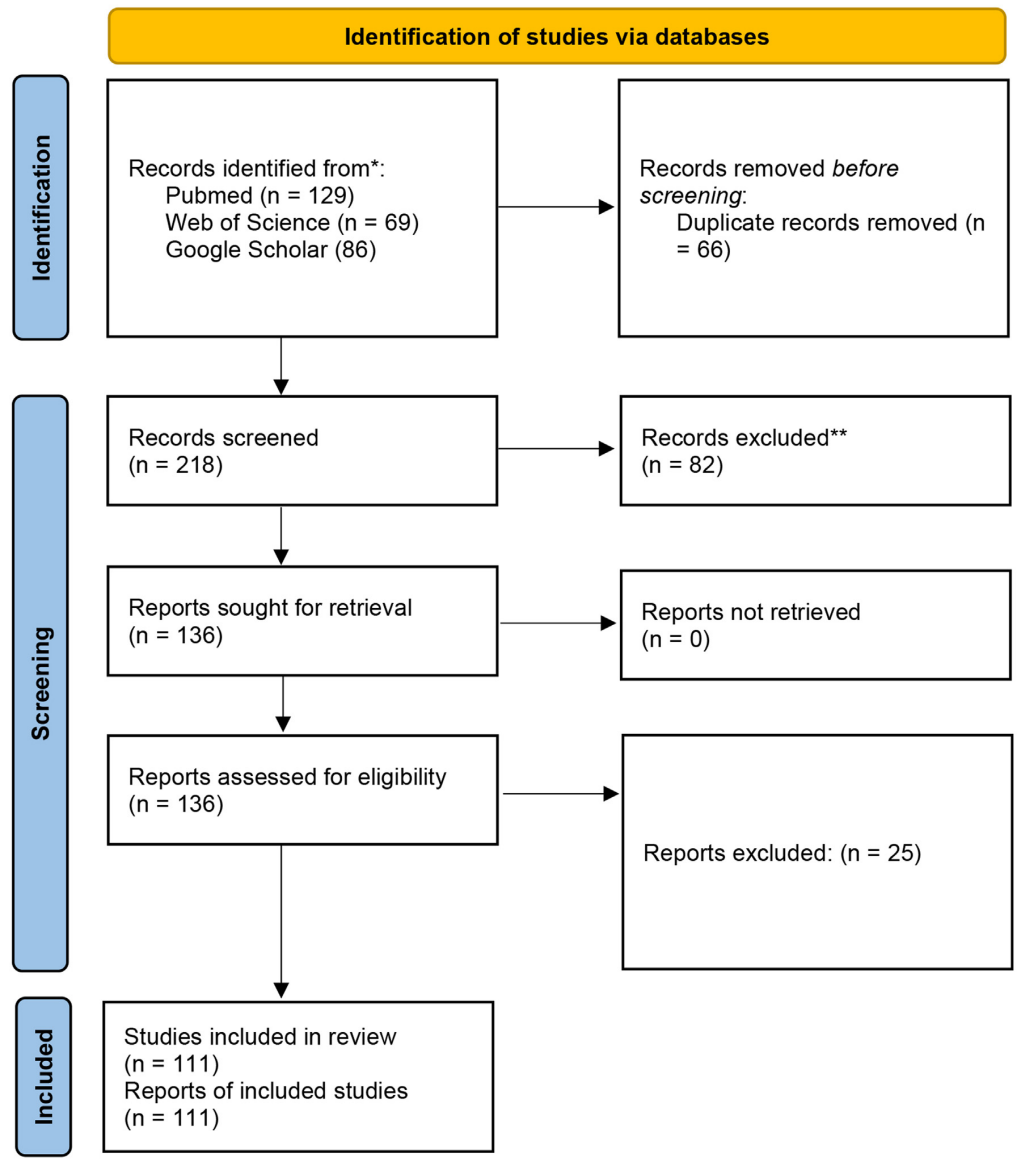
PRISMA flow diagram of the screening and selection process for evidence-based performance and application of AI solutions.

Figure 5 depicts the PRISMA flowchart for Phase 2. A total of 284 records were identified across databases (PubMed = 129, Web of Science = 69, Google Scholar = 86). After removal of 66 duplicates, 218 records underwent title and abstract screening, resulting in the exclusion of 82 articles. One hundred thirty-six full-text articles were assessed for eligibility, and 25 were excluded due to irrelevance. Ultimately, 111 studies met the inclusion criteria and were incorporated into the review.

#### Peer-reviewed evidence of FDA solutions

Table 4 presents the number of peer-reviewed publications for each FDA-cleared dental AI platform, including the breakdown of studies evaluating analytic accuracy/reliability and clinical applications.^68^, ^69^, ^70^, ^71^, ^72^, ^73^, ^74^, ^75^, ^76^, ^77^, ^78^, ^79^, ^80^, ^81^, ^82^, ^83^, ^84^, ^85^, ^86^, ^87^, ^88^, ^89^, ^90^, ^91^, ^92^, ^93^, ^94^, ^95^, ^96^, ^97^, ^98^, ^99^, ^100^, ^101^, ^102^, ^103^, ^104^, ^105^, ^106^, ^107^, ^108^, ^109^, ^110^, ^111^, ^112^, ^113^, ^114^, ^115^, ^116^, ^117^, ^118^, ^119^, ^120^, ^121^, ^122^, ^123^, ^124^, ^125^, ^126^, ^127^, ^128^, ^129^, ^130^, ^131^, ^132^, ^133^, ^134^, ^135^, ^136^, ^137^, ^138^, ^139^, ^140^, ^141^, ^142^, ^143^, ^144^, ^145^, ^146^, ^147^, ^148^, ^149^, ^150^, ^151^, ^152^, ^153^, ^154^, ^155^, ^156^, ^157^, ^158^, ^159^, ^160^, ^161^, ^162^, ^163^, ^164^, ^165^, ^166^, ^167^, ^168^, ^169^, ^170^, ^171^, ^172^, ^173^, ^174^, ^175^, ^176^, ^177^, ^178^ Among FDA-cleared dental AI platforms, Relu Creator and WebCeph were supported by the most substantial body of peer-reviewed evidence. Relu Creator was the subject of 35 publications, with 30 directly evaluating accuracy and/or reliability,^142^, ^143^, ^144^, ^145^, ^146^, ^147^, ^148^, ^149^, ^150^, ^151^, ^152^, ^153^, ^154^, ^155^, ^156^, ^157^, ^158^, ^159^, ^160^, ^161^, ^162^, ^163^, ^164^, ^165^, ^166^, ^167^, ^168^, ^169^, ^170^, ^171^ and 5 for clinical utility.^172^, ^173^, ^174^, ^175^, ^176^ WebCeph followed closely with 33 peer-reviewed publications, including 19 focused on diagnostic accuracy and/or reliability,^68^, ^69^, ^70^, ^71^, ^72^, ^73^, ^74^, ^75^, ^76^, ^77^, ^78^, ^79^, ^80^, ^81^, ^82^, ^83^, ^84^, ^85^, ^86^ and 14 reporting on clinical applications.^87^, ^88^, ^89^, ^90^, ^91^, ^92^, ^93^, ^94^, ^95^, ^96^, ^97^, ^98^, ^99^, ^100^ These studies consistently documented strong agreement with manual cephalometric analysis and broad use in both research and clinical orthodontics.

**Table 4:** Evidence assessing performance and clinical application of FDA-authorized dental AI products.

No.	Company	Product(s) / module(s)	No. of publications (performance + application only)	Performance evaluation	AI application only	Summary of main findings
1	Adravision	Adravision Perio	0	0	0	Nil
2	AssembleCircle	WebCeph	33 (19 + 14)	68, 69, 70, 71, 72, 73, 74, 75, 76, 77, 78, 79, 80, 81, 82, 83, 84, 85, 86	87, 88, 89, 90, 91, 92, 93, 94, 95, 96, 97, 98, 99, 100	Demonstrates strong agreement with manual cephalometric analysis for most landmarks, though some points may require manual review in complex skeletal cases. The platform is widely used in orthodontic research and clinical applications worldwide, supporting planning, growth analysis, and outcome assessment across diverse populations.
3	Audax	WeDoCeph	15 (6 + 9)	76, 79, 85, 101, 102, 103	104, 105, 106, 107, 108, 109, 110, 111, 112	Delivers highly reliable automated cephalometric tracing with strong concordance to manual methods and other commercial AI tools. While landmark-specific deviations may occasionally occur, especially for points like Gonion or Porion, the platform is validated for both routine and complex craniofacial evaluations in skeletal and airway research.
4	Better diagnostics	Better diagnostics caries assist (BDCA) Version 1.0	0	0	0	Nil
5	Cube click	Smile Dx	0	0	0	Nil
6	Dental monitoring SAS	DentalMonitoring	14 (7 + 7)	113, 114, 115, 116, 117, 118, 119	120, 121, 122, 123, 124, 125, 126	Provides clinically accurate 3D models for tooth tracking and offers moderate reliability for oral hygiene assessment. The system is effective for remote orthodontic and periodontal monitoring, supporting reduced appointments, patient engagement, and improved oral hygiene, though results can be influenced by patient compliance.
7	Denti.AI	Denti.AI detect	5 (5 + 0)	127, 128, 129, 130, 131	0	Offers clinically acceptable accuracy for detecting alveolar bone loss and apical radiolucencies in intraoral radiographs. It is most effective as a supportive diagnostic aid for general dentists, especially in periapical images, but is less suitable as a replacement for expert judgment in all settings.
		Denti.AI Auto-Chart	2 (2 + 0)	132, 133	0	Enables accurate, near expert-level detection, classification, and numbering of dental structures in panoramic radiographs, particularly for implants and metal-based restorations. It streamlines radiographic interpretation and automates dental charting, though performance is reduced for less radiopaque materials.
8	ORCA Dental AI	CephX cephalometric analysis	10 (8 + 2)	76, 79, 82, 85, 134, 135, 136, 137	138, 139	Offers reliable, reproducible automated cephalometric analysis with strong agreement to manual tracings and notable efficiency gains. Some parameters may still require manual correction, but overall, the platform is robust and suitable for clinical use, especially with expert oversight.
9	Overjet	Dental assist	0	0	0	Nil
		Caries assist	0	0	0	Nil
		Caries assist	0	0	0	Nil
		Caries assist - pediatric	0	0	0	Nil
		Calculus assist	0	0	0	Nil
		Periapical radiolucency assist	0	0	0	Nil
		Charting assist	0	0	0	Nil
		Image enhancement assist	0	0	0	Nil
10	Pearl	Second opinion	2 (2 + 0)	140, 141	0	Provides high sensitivity and specificity for identifying bone loss, caries, periapical lesions, and restorations on intraoral radiographs. It consistently improves diagnostic accuracy and performance across dentists of varying experience, serving as a valuable adjunct in clinical diagnostics.
		Second opinion CS	0	0	0	Nil
		Second opinion CC	0	0	0	Nil
		Second opinion pediatric	0	0	0	Nil
		Second opinion BLE	0	0	0	Nil
		Second opinion 3D	0	0	0	Nil
		Second opinion periapical radiolucency contours	0	0	0	Nil
11	Relu	Relu creator	35 (30 + 5)	142, 143, 144, 145, 146, 147, 148, 149, 150, 151, 152, 153, 154, 155, 156, 157, 158, 159, 160, 161, 162, 163, 164, 165, 166, 167, 168, 169, 170, 171	172, 173, 174, 175, 176	Clinically validated for expert-level automated segmentation and multimodal registration in 3D CBCT imaging, enabling rapid and reproducible generation of digital dental models for implant planning, orthodontics, and surgery. It dramatically reduces manual workload but is not indicated for 2D radiography.
12	Velmeni	Velmeni for dentists (V4D)	2 (2 + 0)	177, 178	0	Achieves strong correlation with expert radiologists for automated detection of caries, implants, prostheses, and missing teeth on periapical and panoramic radiographs. It reliably handles artifacts and distortions, supporting efficient, reproducible, and operator-independent radiographic diagnosis and treatment planning.
13	VideaHealth	Videa dental assist	0	0	0	Nil
		Videa perio assist	0	0	0	Nil
		Videa caries assist	0	0	0	Nil

Cephalometric analysis solutions such as AudaxCeph and CephX were also well validated, with 6 of 15,^76^^,^^79^^,^^85^^,^^101^, ^102^, ^103^ and 8 of 10 publications,^76^^,^^79^^,^^82^^,^^85^^,^^134^^,^^135^, ^136^, ^137^ respectively, assessing analytic performance. These studies confirmed high concordance with expert manual tracings and robust reproducibility, though some landmarks may require occasional manual review.

DentalMonitoring was moderately well-represented, with 14 total publications,^113^, ^114^, ^115^, ^116^, ^117^, ^118^, ^119^, ^120^, ^121^, ^122^, ^123^, ^124^, ^125^, ^126^ half of which evaluated technical or clinical performance, 113, 114, 115, 116, 117, 118, 119 highlighting effective use in remote orthodontic monitoring. Denti.AI modules also had peer-reviewed support: all five studies for Denti.AI Detect^127^, ^128^, ^129^, ^130^, ^131^ and both for Denti.AI Auto-Chart^132^^,^^133^ directly evaluated diagnostic or analytic accuracy, confirming clinically acceptable results for bone loss, radiolucencies, and automated dental charting.

In contrast, most other platforms, including Better Diagnostics Caries Assist, Cube Click Smile Dx, and all modules from Overjet and VideaHealth, had no peer-reviewed publications identified for either technical performance or clinical application at the time of review. Pearl Second Opinion^140^^,^^141^ and Velmeni for Dentists (V4D)^177^^,^^178^ each had two accuracy studies, supporting their clinical utility. In addition, all other Pearl submodules (Second Opinion CC, CS, BLE, 3D, periapical radiolucency contours, pediatric) lacked any peer-reviewed evidence.

### Commercially available non–FDA-cleared dental AI solutions

Although this narrative review primarily aimed to identify and characterize FDA-cleared dental AI systems, several commercially marketed, cloud-based platforms without verified FDA clearance were also encountered during the screening process. All identified entries were cross-checked against official FDA databases, however, a subset of marketed tools lacked verified clearance despite active deployment.

Table 5 summarizes these non-FDA-cleared platforms, providing supplementary context on the broader adoption of dental AI technologies beyond FDA oversight. It should be emphasized that this list is illustrative rather than exhaustive, intended to enhance transparency rather than expand the study’s scope. This overview highlights the growing commercialization of AI applications in dentistry that, while widely accessible and cloud-deployable, have not obtained confirmed FDA clearance as of July 2025. Many of these systems demonstrated functional overlap with FDA-cleared counterparts, particularly in radiographic interpretation, 3D segmentation, and cephalometric assessment. Collectively, these examples reveal a regulatory and market asymmetry, where numerous AI systems are globally deployed and relatively few have obtained formal FDA authorization.

**Table 5:** Commercially available dental AI platforms without verified FDA clearance.

Sr. no.	Company (Product)	Website URL	Headquarters / Country	Core AI function
1	Diagnocat (Diagnocat AI)	diagnocat.com	United States / Israel	AI analysis of dental radiographs and CBCT for automated detection, segmentation, and diagnostic reporting.
2	CranioCatch (CranioCatch Clinic)	craniocatch.com	Türkiye	AI analysis of dental radiographs and CBCT for automatic detection, segmentation, and diagnostic reporting.
3	Align Technology (Align X-ray Insights; formerly DentalXrai GmbH)	alignxrayinsights.com	United States (acquired DentalXrai GmbH, Germany in 2022)	AI-powered analysis of 2D dental radiographs (panoramic, bitewing, periapical) for automatic detection of caries, periapical radiolucencies, and periodontal bone loss, with colour-coded annotations and standardized diagnostic reports.
4	DeepCare (Multimodal Dental AI)	deepcare.com	China	AI analysis of dental radiographs and CBCT for automated detection, segmentation, and diagnostic reporting.
5	Eyes of AI (EAI Detect / Trace / Segment)	eyesofai.com	Australia	AI analysis of 2D dental radiographs and CBCT for automated detection, tracing of cephalometric landmarks, segmentation, and diagnostic/reporting support.
6	Allisone (Allisone Platform)	allisone.ai	France	AI-powered visualization and patient-education tool that highlights lesions on dental radiographs with color coding, generates annotated reports, and helps increase patient understanding and treatment plan acceptance.
7	CoTreat (CoTreat Navigator)	cotreat.ai	Australia	AI-powered analysis of dental practice data (imaging and records) for automated detection of missed treatment opportunities, treatment-plan presentation, and business-growth insights.
8	scanO (scanO AI Platform)	scanoai.com	India	AI-powered oral health ecosystem offering rapid self-scans and clinic tools for automated detection of 40+ dental conditions, simplified workflows and instant reports.
9	DDH (Ceppro)	ddhinc.net	South Korea	AI-powered cephalometric and panoramic dental imaging tool for automated landmark detection, measurement, superimposition and report generation.
10	Dentem (Dx Vision)	dentem.co	Canada	AI-powered dental practice software with an integrated algorithm (Dx Vision) for detection of issues in dental X-rays (eg, caries, bone loss) and automated reporting.
11	DentiBird (Dentbird Platform)	dentbird.com	South Korea	AI-powered web-based CAD software that automates crown and prosthesis design using intraoral scan data.
12	Promaton (Promaton Platform)	promaton.com	Netherlands	AI analysis of dental CBCT and intraoral scans for automatic segmentation, treatment planning (implantology, prosthetics, orthodontics), and 3D model generation.
13	Smilo.ai (Smilo.ai App)	smilo.ai	India	AI-powered virtual dental platform for smartphone selfies: instant oral-health screening (cavities/gum disease), smile simulation, remote triage and patient engagement.
14	Smart Dent (Smart Dent Platform)	smartdent.tech	Iran/Canada	AI-powered platform for dental image analysis and workflow automation (including diagnosis assistance, risk assessment, and report generation).
15	OraQ (OraQ Clinical DSS)	oraq.ai	Canada	AI-based clinical decision support system for dental practices that analyzes medical history, exam data and imaging to generate personalized risk profiles and treatment recommendations.

Among the platforms, Diagnocat (Diagnocat Inc.) stands out as a Class II medical image analyzer listed in the FDA database for export-only use, indicating registration without 510(k) authorization for clinical deployment within the United States. Similarly, CranioCatch (CranioCatch Inc.), Promaton (Promaton B.V.), and several other platforms demonstrate compliance with international quality management or regional certification standards, yet lack publicly verifiable evidence of FDA marketing authorization. These systems are deployed for clinical research and/or regional dental practices, offering functionalities such as automatic detection of pathologies, segmentation of dental structures, treatment planning, and cephalometric analysis. These platforms illustrate how non-FDA-cleared AI systems are contributing to the global diffusion of dental AI technologies, supported by cloud-based accessibility and rapid commercial scalability. While their growing use highlights the technological maturity and international reach of AI in dentistry, it also highlights a regulatory gap between innovation, validation, and formal approval, emphasizing the need for harmonized international standards in AI-driven dentistry.

## Discussion

The emergence of standalone, FDA-cleared, AI platforms for dental imaging represents a pivotal shift in the trajectory of digital dentistry. These platforms show a dedicated, scalable, and clinically validated approach to AI-driven dental imaging tasks. This transition has been propelled by advances in ML, the availability of large annotated datasets, and evolving regulatory frameworks that encourage robust validation.^179^ As a result, dental imaging is increasingly benefiting from the same technological rigor and regulatory oversight that have reshaped medical radiology,^180^ with AI now positioned at the core of image interpretation, diagnostic planning, and treatment monitoring.

This review found that almost all standalone dental AI platforms received FDA clearance through the 510(k) pathway, classifying them as moderate-risk medical devices. The 510(k) process relies on comparing new products to existing, already-cleared devices (predicates) and is designed to speed up market entry for technologies deemed substantially equivalent.^181^ While this approach has allowed dental AI platforms to become available relatively quickly, it raises questions about whether current standards are well-suited for evaluating the unique and evolving nature of AI, which may perform differently as algorithms or data change over time. To organize and categorize these devices, the FDA assigns product codes based on the main function of the software,^182^ such as MYN for caries detection or QIH for general dental imaging. These codes are useful for standard, single-purpose tools. However, many new dental AI platforms now offer a wide range of features and functions within a single system. As a result, these broad product codes may not fully describe the multifunctional capabilities of modern AI platforms,^183^ making it more difficult for clinicians and regulators to assess and compare products based solely on these classifications.

Furthermore, this review identified only one standalone dental AI platform that was cleared via the De Novo pathway, a regulatory route intended for entirely new types of devices without clear predicates.^184^ The predominance of the 510(k) pathway suggests that regulatory processes may still favor incremental innovation based on previous products, rather than supporting the introduction of truly novel or transformative technologies. As dental AI platforms continue to become more complex and versatile, there will be a need for the regulatory system to adapt, ensuring that safety and performance are properly evaluated while still enabling timely access to innovation. Ongoing collaboration among developers, regulators, and clinicians will be essential to keep regulatory standards up to date with the rapid evolution of dental imaging AI.

The progression of AI platforms was observed from single-purpose detection tools to comprehensive multi-purpose assistants, which reflects both technological maturation and shifting clinical expectations. The inclusion of pediatric modules, CBCT segmentation, multi-condition detection, and automated charting illustrates how industry and regulators have responded to the demand for versatile solutions capable of addressing a broad range of dental conditions within a single platform. While this expansion holds promise for greater efficiency and improved patient management, it also raises new considerations regarding the integration of complex AI systems into routine clinical workflows. As platforms increasingly cover multiple tasks and modalities, there is a risk that “one-size-fits-all” approaches may overlook specific nuances of certain conditions or patient groups.^185^ Therefore, future research should focus on assessing not only the aggregate performance of these platforms, but also their utility and accuracy across diverse clinical scenarios.

While the platforms reviewed generally met or exceeded regulatory benchmarks for accuracy, sensitivity, and specificity at the time of clearance, the real-world significance of these metrics remains uncertain. AI systems in medical imaging are normally validated using retrospective datasets and manufacturer-reported outcomes, often without multi-center clinical trials to confirm generalizability.^186^ This dependence on narrowly defined evidence limits understanding of how AI tools perform under everyday clinical variability, differences in patient demographics, imaging conditions, and workflow contexts. The resulting gap between regulatory approval and clinical performance highlights the need for continuous, independent assessments that examine how AI behaves in real-world dental practice. Only through such validation can clinicians gain confidence in integrating AI-driven decision support safely and effectively into patient care.

A critical challenge facing dental AI is algorithmic bias stemming from nonrepresentative training data and model design choices.^187^ When datasets underrepresent certain age groups, ethnicities, anatomical variations, or imaging protocols, performance may vary across populations, producing systematic errors that compromise diagnostic accuracy and equity.^188^ Bias may also arise from architectural decisions, inherited bias from pretrained backbones, and feedback loops during clinical deployment.^189^ Schwendicke et al.^190^ highlighted that both dataset composition and reporting practices can perpetuate bias, recommending heterogeneous data, independent test sets, and subgroup-level reporting (eg, by patient risk or data source) to detect and mitigate such effects throughout study design and evaluation. Ethical frameworks further advocate prospective drift monitoring, transparent subgroup performance disclosure, and algorithmic safeguards to prevent bias amplification.^191^ As AI becomes more deeply embedded in dental care, ensuring demographic and clinical fairness must remain a cornerstone of both model development and postmarket surveillance.^192^

Beyond these design and data-related concerns, a related limitation is the scarcity of independent, external validation studies. While regulatory clearance verifies that a system meets internal performance thresholds, it does not ensure consistent behavior across diverse clinical settings or over time.^179^ The absence of standardized evaluation frameworks and the persistence of single-source or manufacturer-reported data introduce blind spots that compromise generalizability.^193^^,^^194^ Addressing this, the DentalCOMS initiative (Büttner et al.)^195^ has proposed structured outcome reporting standards to enhance transparency, comparability, and methodological rigor in dental AI research. These guidelines reflect a broader shift toward evidence-based verification of clinical utility, emphasizing that models must be tested under heterogeneous conditions and continuously monitored after deployment. Future research should therefore prioritize prospective, multi-center trials that evaluate not only accuracy but also robustness, usability, and long-term stability. Encouragingly, professional organizations such as the American Dental Association also stress independent dataset validation and ongoing performance tracking as prerequisites for the safe, equitable, and sustainable adoption of AI in dentistry.^196^

Despite high performance metrics observed for the platforms included in the review, several persistent barriers may impede the successful integration of standalone AI platforms into everyday dental practice. The lack of systematic postmarket surveillance means that potential issues such as model drift, evolving population demographics, or changes in imaging protocols may go undetected, potentially compromising patient safety and outcome reliability over time.^197^ Another major barrier is interoperability, where many dental clinics use different brands and models of imaging equipment and electronic health records, but there are currently no widely accepted standards to ensure that AI platforms can easily communicate and work with all of these systems.^198^ This lack of compatibility can make it difficult for practices to adopt AI tools smoothly, limiting their effectiveness and slowing widespread implementation. Furthermore, the risk of algorithmic bias stemming from nonrepresentative training datasets poses the danger of uneven accuracy across different demographic or clinical subgroups, thereby threatening equity in patient care.^199^ Finally, the real-world implications of AI-generated false positives or negatives are not yet fully understood, underscoring the necessity for robust human oversight, clear accountability frameworks, and ongoing clinician education to ensure that AI enhances, rather than undermines, clinical decision-making.^200^

Nevertheless, thoughtfully implemented AI platforms hold significant potential for improving workflow consistency, efficiency, and access to high-quality care. Features such as automated charting, multi-condition diagnostics, and patient monitoring can streamline practice operations and enhance patient engagement. However, realizing these benefits require not only technological adoption but also workflow redesign, ongoing clinician training, and strong governance to prevent overreliance on AI and to safeguard patient outcomes. Transparent reporting of platform performance, continuous software improvement, and sustained collaboration among developers, clinicians, and regulators will be vital to address emerging ethical, legal, and practical challenges.^201^

This review had several limitations. First, it was restricted to standalone, cloud-based FDA-cleared AI platforms for dental imaging, deliberately excluding traditional commercial dental software suites with embedded or adjunctive AI modules. This choice was made because standalone platforms undergo independent regulatory review as distinct medical devices, with clearly defined indications, performance metrics, and clinical validation. In contrast, traditional dental software suites may integrate AI functionalities as ancillary features without undergoing separate FDA evaluation, making it difficult to assess their safety, effectiveness, and real-world clinical impact in a standardized way. Focusing on independently regulated platforms ensured that the review was clinically and regulatory relevant to decision-makers and practitioners seeking solutions with proven safety and efficacy for patient care. Second, there was considerable variation in the amount and quality of independent clinical evidence supporting the included platforms. While some products had strong external validation, many relied mainly on regulatory or manufacturer-reported data. This highlights the need for more independent, multi-center studies to confirm their real-world performance. Third, only platforms approved by the FDA were included. This approach was chosen because the FDA offers uniquely comprehensive, detailed, and publicly accessible regulatory data, enabling transparent and standardized reporting across products. In contrast, equivalent regulatory information for dental AI platforms approved in other regions such as Europe, Japan, China, or South Korea, are often not as readily available, consistently detailed, or standardized for independent review. As a result, focusing on FDA-cleared products helped ensure methodological rigor and data reliability, but may have excluded important international innovations. Finally, a narrative review methodology was employed, as it allows for a more flexible and contextual synthesis of regulatory, technical, and clinical information across this rapidly evolving field. However, this approach might have also introduce selection and reporting bias.

## Future recommendations

To maximize the potential of AI platforms in dental imaging, several key priorities should guide future research and clinical adoption. First, independent, prospective, and multi-center studies are urgently needed to confirm the effectiveness, reliability, and safety of these tools across diverse clinical environments. Establishing universal interoperability standards will be essential to facilitate integration with existing dental imaging systems and electronic health records, supporting broad-based adoption and clinical utility. Continuous auditing and transparent reporting of algorithmic performance, including subgroup analyses by age, gender, and imaging modality are needed to identify and mitigate potential bias. In addition, standardized benchmarks for clinical validation and cross-platform comparison will enable clinicians to make evidence-based decisions regarding AI adoption. Finally, sustained collaboration among AI developers, regulators, clinicians, and researchers will be crucial to ensure that these technologies deliver on their promise of improved accuracy, workflow efficiency, and equitable patient care.

## Conclusion

The standalone AI platforms for dental imaging represent a paradigm shift in digital dentistry, offering autonomous and validated support for diagnosis, treatment planning, and patient monitoring across diverse imaging modalities and patient populations. FDA clearance distinguishes these platforms as clinically validated medical devices, setting a benchmark for safety and efficacy in an emerging field dominated by experimental algorithms and unregulated modules. By systematically mapping the current portfolio of regulatory-approved dental AI technologies, this review delivers an accessible scientific reference for clinicians, enabling informed decision-making and promoting evidence-based integration of AI into routine dental care.

## Author contributions

Sohaib Shujaat –Conceptualization, methodology, literature search, screening and selection, data extraction, investigation, data curation, writing –original draft, writing –review and editing, supervision, project administration. Hend Aljadaan –Literature search, screening and selection, data extraction, investigation, data curation, writing –review and editing. Hessah Alrashid –Investigation, writing –review and editing. Ali Anwar Aboalela –writing –review and editing. Marryam Riaz –Validation (adjudication of discrepancies), writing –original draft, writing –review and editing.

## Funding

This research did not receive any specific grant from funding agencies in the public, commercial, or not-for-profit sectors.

## Conflicts of interest

None disclosed.
